# Exploring the Determinants Impacting Work–Life Balance for General Nurses in Diverse Healthcare Settings: A Cross-Sectional Study

**DOI:** 10.1155/jonm/8856776

**Published:** 2025-07-08

**Authors:** Aneta Hujová, Miroslava Zemanová, Daša Stupková, Michal Vostrý

**Affiliations:** ^1^Department of Nursing, Jan Evangelista Purkyně University in Ústi nad Labem, Ústí nad Labem, Ústí nad Labem Region, Czech Republic; ^2^Department of Specific Health Disciplines, Jan Evangelista Purkyně University in Ústi nad Labem, Ústí nad Labem, Ústí nad Labem Region, Czech Republic; ^3^Department of Nursing, Slovak Medical University in Bratislava, Bratislava, Slovakia; ^4^Department of Occupational Therapy, Jan Evangelista Purkyně University in Ústi nad Labem, Ústí nad Labem, Ústí nad Labem Region, Czech Republic

**Keywords:** balance, factors, family, general nurses, work

## Abstract

**Background:** Work–life balance (WLB) is a crucial factor influencing the well-being and job satisfaction of healthcare professionals, particularly general nurses. Increasing job demands, shift work and inadequate remuneration contribute to WLB challenges, often leading to burnout, stress and nurse turnover. Understanding the factors impacting WLB can guide improvements in workplace policies and healthcare outcomes.

**Aims:** This study aims to evaluate the WLB of general nurses in the Czech Republic and examine the influence of demographic and professional factors, such as length of experience, workplace type, educational attainment and family structure, on WLB.

**Methods:** A cross-sectional study was conducted from April to September 2024, involving 200 general nurses. Data were collected through a demographic questionnaire and the Work–Family Conflict Scale, which measures work-to-family and family-to-work conflict on a 4-point Likert scale. Descriptive statistics and inferential analyses, including chi-squared and Spearman correlation tests, were used to assess the relationships between WLB and selected variables.

**Results:** The findings revealed moderate levels of work–family conflict, with higher scores for work negatively impacting family life compared to the reverse. Significant correlations were found between WLB and factors such as workplace type and educational attainment, with nurses in intensive care units and those with secondary education reporting higher conflict. Surprisingly, no significant relationship was observed between WLB and the number of children or shift work. Older nurses and those with longer professional experience reported lower levels of work–family conflict.

**Conclusion:** The study highlights the importance of addressing workplace factors, such as workload and professional development opportunities, to improve WLB among general nurses. Promoting WLB can reduce stress and burnout while enhancing job satisfaction and care quality. Further research is needed to explore individual needs and the potential for personalised scheduling in nursing practice.

## 1. Introduction

According to the European Labour Authority [1], an average person spends approximately one-third of their life at work. Therefore, it is necessary to find ways to ensure an optimum balance between work and private life. The work–life balance (WLB) is defined as a relationship between work and nonwork activities that is balanced and the demands of both spheres are equal [2]. This means that a human can experience satisfaction in various aspects of their life and achieves an optimum balance between their work and private life [1, 3]. Work–family conflict is a type of interrole conflict that arises when work and family commitments are incompatible or contradictory in a certain respect [4].

WLB is an essential factor contributing to the well-being of employees at various job positions [5] and is essential for maintaining emotional and mental well-being; however, it is being lost due to increasing job demands and stress exposure. This imbalance can lead to increased stress, depression and even burnout syndrome. The prevention is to observe leisure time between work during which individual should not think about work responsibilities [6]. As stated by Tanaka et al. [7], work–life imbalance negatively affects job satisfaction, stress management and life satisfaction.

The European Agency for Safety and Health at Work states that a work–life imbalance is one of the key psychosocial factors at the workplace [8]. This issue is especially relevant to health professionals facing stressful situations, overwork due to understaffing or feelings of underappreciation [9]. Work–life conflict may be aggravated further by long working hours, shift operation, irregular working hours and insufficient financial remuneration [2, 10]. At the same time, these negative aspects contribute to nurse turnover in the health sector, leading to a persistent shortage of health personnel, which is exacerbated by this [11].

Reference [12] states that the shortage of qualified nurses is a global problem, exacerbated by the difficulty of integrating nurses into individual workplaces. In 2020, there was a shortage of approximately 5.9 million nurses and according to the World Health Organization, by 2030, there will be a shortage of nearly 14 million health workers [13]. In addition, nurses work in shift patterns that lead to irregular life rhythms, which can lead to imbalances [7].

In order to retain new staff, it is important to identify the reasons for turnover on the ward. Primarily, it is the search for better working conditions, a higher standard of living and the opportunity for professional development [12].

Just an effective WLB is an important preventive factor against burnout [5]. On the contrary, deterioration of this balance may have adverse effects on the life and behaviour of health professionals [2] and compromise the quality of health care provided. It is assumed that a long-term work–life imbalance contributes to burnout [3]. Every person may perceive the WLB differently, but it is essential that this balance should make it possible for them to pursue their interests, maintain social connections and improve work results [1]. WLB is necessary for the satisfaction of health professionals with the working conditions, which contributes to minimising stress and securing workplace well-being [14]. Improved health and well-being at the workplace are aided by a pleasant and motivating working environment where one can better combine work and family commitments, thereby achieving WLB [3, 4].

At the same time, nurses' health has been shown to impact patient care [15].

Reference [4] points out that women are more susceptible to greater work–family conflict, especially owing to increasing employment and roles in family life. This study examines the WLB of general nurses (GNs), which is relevant considering the fact that up to 98% of this profession is practised by females according to [16].

## 2. Objective

The objective of the study is to explore and analyse the determinants influencing the WLB of GNs working in healthcare facilities in the Czech Republic. The study focuses on comparing the impact of demographical and professional factors on the WLB, as understanding the factors that influence this balance is key to ensuring quality care [17].

## 3. Materials and Methods

### 3.1. Study Design

The research was conducted as a cross-sectional study in accordance with the STROBE guidelines [18] commenced in April 2024 under the auspices of the Internal Grant Agency of the Faculty of Health Studies of the J E Purkyně University, project No. 72141_16_2038_01 titled ‘The quality of work life of general nurses'.

### 3.2. Participants and Sampling

A purpose (intentional) sampling method was used, focussing exclusively on GNs. The recruitment process was clearly communicated to potential participants through posters displayed in selected healthcare facilities, as well as personal invitations sent out through professional nursing networks and social media platforms, and was also reiterated in the introduction of the questionnaire. Participation was entirely voluntary. This approach was chosen to specifically target the professional group relevant to the research objective. Only fully completed questionnaires were included in the final analysis.

The study included 200 GNs who had met the conditions laid down in Act No. 96/2004 [19], had at least a year's nursing experience, had no language barrier and worked in healthcare facilities in the Czech Republic. Respondents not meeting these criteria were not included in the study. The questionnaire survey was anonymous and voluntary; by completing it the study participants expressed their voluntary consent to the processing of the results. The questionnaires were distributed through direct links or QR codes and filled in on the Survio platform.

Data were collected through a validated questionnaire with demonstrated reliability. Although the sample was not randomly selected, its composition faithfully reflects the structure of the target population of GNs in the Czech Republic, which supports the generalisability of the findings.

To assess possible selection bias, the average age of respondents was compared with data from official statistics on the age structure of nonmedical workers in the Czech Republic. As the data were collected exclusively online, the risk of selection bias could not be excluded, especially with regard to the age, technical accessibility and computer literacy of the participants. For this reason, the basic sociodemographic characteristics of the sample were compared with available data on the general population of nurses in the Czech Republic to assess the level of representativeness of the sample. In 2023, there were 78,012 registered GNs in the Czech Republic [20]. The mean age of this population in the same year was 48.2 years, suggesting a possible slight selection bias in favour of younger respondents in the study [21].

### 3.3. Data Collection Tools

The data were obtained by means of two tools: a demographical questionnaire and a standardised questionnaire. The demographical questionnaire gathered basic demographical and professional information on the respondents. It contained a total of 10 questions focussing on age, gender, marital status, number of children, length of nursing experience, shift operation, workload, workplace, highest education attained and continuing education of the respondents. The Work–Family Conflict Scale [4] was used to evaluate WLB. This four-item scale measures the bidirectional process of work–family conflict and has been used since 2002 in the International Social Survey Programme (ISSP), specifically in the ‘The Family and Changing Gender Roles' module [22], having been translated into more than 30 languages including Czech. The choice of this scale was mainly motivated by its international comparability, brevity and time-efficiency, which is important in research among health professionals who often face a heavy workload. For this reason, this four-item scale was chosen, which has been widely used in international research and validated in many cultural contexts. Cronbach's alpha values typically range from 0.50 to 0.85 across countries, reflecting the expected variability in short measurement instruments. In the Czech context, the reliability of the scale was confirmed by a Cronbach's alpha value of 0.78, which is relatively high compared to values reported in other countries (e.g., in Slovakia, α = 0.82; in Germany, α = 0.72; in Austria, α = 0.67). Although this is a short scale, its internal consistency was considered sufficient for research on work–family balance among Czech GNs [4].

The respondents gave their answers on a 4-point scale, assessing the frequency of situations that had happened to them over the last 3 months: 4 = several times a week, 3 = several times a month, 2 = once or twice and 1 = never.

The first two items seek to ascertain the interference of work on family life:

• I came home from work so tired that I was not able to do the things at home that were necessary (Item 1).
• *Because of the amount of time I spent at work, it was difficult for me to fulfil family commitments* (Item 2).

The next two items investigate the interference of family life on work:

• *Owing to work in the household I had to do, I arrived at work so tired that I was not able to work well* (Item 3).
• *I have found out that because of family commitments it is difficult for me to focus on work* (Item 4).

The answers were converted into a score (never = 1; several times a week = 4), with a higher score meaning greater work–family conflict.

### 3.4. Pilot Testing

A pilot study took place prior to the main collection of data to verify comprehensibility and suitability of the questionnaires, during which 10 questionnaires were distributed and 100% of them returned; these answers have not been included in the main study. The data collection took place from April to September 2024, and 200 GNs took part.

### 3.5. Research Sample

The research sample comprised 200 GNs working in healthcare facilities in the Czech Republic (descriptive statistics in Table 1). Of whom, 97.5% were females (*n* = 195) and males only accounted for 2.5% (*n* = 5). The average age of the respondents was 45.1 years. The educational attainment was distributed among the participants as follows: secondary (*n* = 93, 46.5%), post-secondary vocational (*n* = 26, 13%), university bachelor's degree (*n* = 51, 25.5%) and university master's degree (*n* = 30, 15%). Only 42 respondents (21%) completed additional education.

Most of the respondents were married (50%, 5%), 15% were divorced, 16.5% were single, 14.5% lived with a partner and four female respondents (2%) were widowed. Almost half the respondents had two children (41%), 25.5% (*n* = 51) of the respondents had one child and 26.5% (*n* = 53) of the respondents had no children. One female respondent had four children and the others had three children (6.5%).

A total of 168 respondents (84%) worked full-time, and 53.5% (*n* = 107) worked in shifts. The length of experience was distributed as follows: 1–5 years (*n* = 23, 11.5%), 6–10 years (*n* = 14, 7%), 11–15 years (*n* = 19, 9.5%), 16–20 years (*n* = 25, 12.5%), 21–30 years (*n* = 66, 33%) and 31 and more years (*n* = 53, 26.5%). Most respondents (*n* = 62, 31%) worked in intensive care units (ICUs), anaesthesiology and resuscitation departments or in intermediary care, then in outpatient departments (*n* = 53, 26.5%) and in standard inpatient departments (*n* = 49, 24.5%). A leading position was occupied by 36 respondents (18%).

### 3.6. Research Questions

• Research question: What are the differences in the WLB among GNs in relation to selected demographical and professional factors (length of experience, workplace, educational attainment and number of children)?
• Descriptive hypothesis: There are statistically significant differences in the WLB that are influenced by selected demographical and professional factors (length of experience, workplace, educational attainment and number of children).

### 3.7. Data Analysis Methods

In this study, we employed descriptive statistics and statistical tests to analyse relationships between selected demographical and professional variables (e.g. length of experience, workplace, educational attainment and number of children) and WLB.

For each variable, we calculated the percentage, median, standard deviation (SD), minimum and maximum, which provided a detailed overview of the distribution and variability of the data. To verify the statistical significance of the differences between the individual categories, we used a chi-squared test. The statistical significance was determined by means of the *p*value, and the results with *p* values lower than 0.05 were considered as statistically significant. Because some variables were ordinal and some did not meet the assumption of a normal distribution, nonparametric tests were chosen as the most appropriate methods of analysis. Multiple linear regression analysis was also performed to control for potential confounding variables such as age, marital status, shift work, length of nursing experience, education and number of children.

In addition, we made use of the Spearman test to ascertain monotonous relationships between all variables, which allowed us to evaluate the strength and direction of correlation between various factors. Descriptive statistics provided the context for data interpretation, including the use of minimum and maximum values to understand the range of the data and the median to describe the central tendency. Repeat participation in the survey link was strictly limited, and there were no missing values, as all questions were answered.

## 4. Results

Table 2 illustrates the distribution of the answers to the individual items of the WLB scale. A total of 37.9% of the respondents stated that they experienced work–family conflict very often, even several times a week. This conflict manifested itself most markedly by the inability to perform home commitments due to fatigue from work, which reflects in the mean score of 3. A slightly milder conflict was recorded in the second question, where 33.2% of the respondents stated that they also experienced an impact of their workload on their family life. As far as the impact of personal life on job performance is concerned, only 8.1% of the respondents admitted that they would arrive at work so tired due to family commitments that they would not be able to work efficiently, and another 6.2% of them said that family commitments affected their ability to focus on work.

Furthermore, relationships between selected demographical and professional factors were examined. Most of the results were not statistically significant, but a significant relationship (*p*=0.027) was ascertained between the level of education attained and the impact of work-related fatigue on the performance of home commitments. Some professional factors approached the limit for statistical significance, e.g., the relationship between the workplace and the inability to perform family commitments owing to time constraints (*p*=0.0943). Also, the relationship between the level of education attained and the assertion that work commitments cause fatigue preventing an efficient job performance was found to be just below the significance limit (*p*=0.051).

Table 3 illustrates level of conflict between selected demographical and professional factors. The mean score was 9.27 (SD 2.36, median 9), with the recorded values ranging from 4 to 16 points. This total score indicates that, in general, the respondents face a medium level of conflict. Both the highest and lowest levels of conflict were identified in respondents who have three and more children. In terms of the type of workplace, the highest level of conflict was stated by respondents working in standard inpatient departments, whereas the lowest level of conflict was recorded in the case of nurses working in outpatient departments. This result is probably connected with the fact that outpatient nurses do not work in shifts, they are in short-term contact with patients and have more specific tasks. Surprisingly, nurses in leading positions stated a higher level of work conflict although their working conditions are very similar to those in outpatient departments; however, this difference may be due to greater responsibility towards the staff and the pressure on the part of the middle and senior management.

The highest level of conflict was registered in respondents having higher vocational education, while none of the nurses possessing university master's education stated a high level of conflict. The lowest score in the high-conflict zone was recorded in nurses having more than 30 years' work experience, which we expected based on research studies.

Table 4 summarises all statistically significant differences (*p* < 0.05) in items assessing work–life conflicts by respondent characteristics. The most significant differences were found for the following groups:

• Workplace: Statistically significant WLB problems were observed especially among critical care workers (*p*=0.0051), who showed a significant burden in fulfilling family responsibilities due to workload. Respondents from standard wards reported impaired concentration due to family commitments (*p*=0.0229). In contrast, outpatient and managerial staff did not show significant differences.
• Number of children: This factor did not show a statistically significant effect on WLB, although a higher burden for parents with more children might be expected. However, some *p* values lie just above the significance level (*p*=0.0934), which may indicate possible trends that should be investigated in future studies.
• Education: Significant differences were found for respondents with secondary education who reported work exhaustion affecting domestic responsibilities (*p*=0.0072) and also a negative impact of domestic commitments on work performance (*p*=0.0141). For the other education groups, the differences were not statistically significant, although the values were close to the significance threshold for master's education (*p*=0.0571), which may indicate weaker but significant relationships.
• Length of experience: A significant effect was confirmed only among respondents with 1–10 years of experience, where family commitments influenced concentration at work (*p*=0.01). No significant relationships were found in more experienced groups (11+ years), although in the group with 31 or more years of experience, the *p* value was close to the significance threshold (*p*=0.0939), which may indicate a potential trend.

Overall, most of the analysed *p* values exceeded the significance level of 0.05, suggesting that the observed factors generally do not explain differences in the perceived WLB. Exceptions include specific groups of intensive care workers, individuals with secondary education and younger healthcare workers, for whom disruption of balance is more pronounced. Marginally significant results may represent important directions for further research.

**Figure 1:**
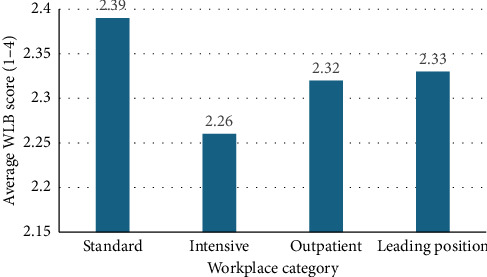
Average work–life balance score by workplace (*N* = 200).

**Figure 2:**
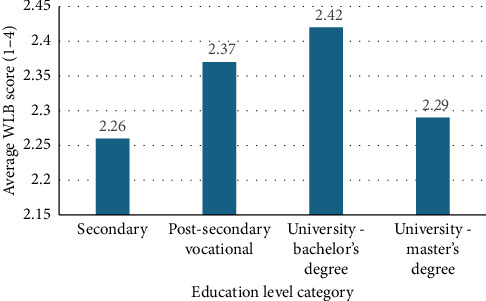
Average work–life balance score by education level.

Figures 1 and 2 illustrate the differences in average WLB scores across different types of workplaces and education levels.

Nurses working in standard hospital wards reported higher levels of work–life conflict compared to nurses working in ICUs or outpatient settings. Interestingly, nurses in managerial positions also reported relatively less conflict, which may be attributed to greater autonomy and control over work schedules.

In terms of education (Figure 2), respondents with only a high school education or a master's degree reported the lowest WLB conflict, while respondents with a bachelor's degree experienced the highest conflict. This could suggest that nurses with a secondary education may face greater work-related demands without adequate decision-making authority or support systems.

Table 5 illustrates the results of the linear regression model that was used to identify predictors of WLB scores. Seven independent variables were included in the model, but none of these showed a statistically significant relationship with the WLB score (*p* values > 0.05). Confidence intervals for all estimates included zero, indicating the absence of a significant effect of the variables on the outcome of interest.

The variable ‘workplace' had the highest estimate (*B* = 0.345), but statistical significance was not reached here either (*p*=0.180). The other predictors had very low effect estimates and wide confidence intervals.

These results suggest that none of the sociodemographic or work characteristics examined showed a significant effect on WLB in the study population.

Spearman's correlation coefficients in Table 6 show that there exist only weak relationships between the respondents' characteristics and various aspects of the work–family conflict, with several exceptions. In general, age has a very weak relationship to all the items under consideration, and the greatest but still insignificant negative correlation (−0.178) is between age and the feeling of fatigue due to home commitments that affect job performance. This relationship indicates that with higher age, the respondents may feel such fatigue less.

Gender has a mild positive relationship (0.1) to the feeling of fatigue due to home commitments affecting job performance, which indicates that females may be slightly more affected by this factor than males. However, marital status does not have a significant correlation with any item under consideration, so it probably does not affect the work–family conflict perceived. Similarly, the number of children shows a weak negative correlation (−0.106) with the feeling of fatigue due to home commitments that affect job performance, which may indicate that a higher number of children weakly reduces this conflict.

Education manifests a weak positive relationship (0.184) with the feeling of fatigue due to home commitments, which indicates that respondents who have achieved higher education may experience a slightly heightened conflict in this area. Additional education also has a very weak relationship to this item (0.087), which means that its influence on the conflict is insignificant. The workload does not manifest any significant correlation with any item, and thus it probably has no influence on the conflicts under consideration.

Shift operation is associated with weak negative correlations with the difficulty in performing home commitments (−0.125) and with the feeling of fatigue due to home commitments that affect job performance (−0.118). This may suggest that shift operation weakly diminishes these conflicts. The length of experience has a slight negative correlation (−0.192) with the feeling of fatigue due to home commitments, which indicates that respondents with a longer work experience may perceive lesser conflict in this area.

The interesting finding is the relationship between the type of workplace and the difficulty in concentrating on work owing to family commitments (0.354). This fairly strong positive relationship indicates that the type of workplace significantly affects the respondents' ability to concentrate at work, with family commitments playing a bigger role here.

## 5. Discussion

This study examined the current WLB situation among GNs working in health care institutions in the Czech Republic. The purpose of this study was to evaluate which of the selected demographical and professional factors impact WLB. Our results showed that WLB is influenced by the following sociodemographic factors: workplace, highest level of education and length of experience. The principal finding of our study was the fact that approximately two-thirds of the respondents had stated that their private life was impacted by their workload. This was mentioned mainly by those respondents with a high school education, who had two children, worked in outpatient facilities and had more than 20 years of nursing experience.

GNs showed mild to moderate conflict, with an overall score of 2.32. This score shows that respondents more frequently reported that they encountered the situation from the questionnaire ‘once or twice' and ‘several times a month'. The score for work–family conflict as a result of a negative impact of work on family life was 2.89. By contrast, the score for family–work conflict as a result of a negative impact of the family on work life was 1.75. Reference [23] looked at the balance between nurses working in psychiatric hospitals. The results showed that nurses (70.3% female) had a mean WLB of 3.1 (SD 0.5) with a range of 1–4, corresponding to the category of sometimes or occasionally. This score shows a relatively high WLB. At the same time, a significant correlation was found between employment and overtime. Overtime and percentage of employment decreased this balance. In contrast, number of employees and adequacy of resources were associated with higher balance. This is confirmed in [24], who identified excessive overtime and undue pressure in the workplace as negative factors.

Our results further show that older respondents face less work–family conflict compared to younger respondents, which is also proven by the study of [25]. This fact may be linked to an unstable position in the social group as well as a lower salary or a higher shift rate; the shift rate may affect their private social life. Reference [26] found a statistically significant association (0.051) between tenure and balance. Respondents aged 31–40 years responded more positively. However, direct comparison of our findings with those of [26] is complicated by differences in shift work organisation. In Czech hospitals, inpatient wards and ICUs have a continuous working pattern, with 12-hour shifts combined with irregular schedules and frequent overtime worked at the employer's behest [27], which may have a different impact on WLB than in other countries. Reference [28] also confirms greater work–family conflict recorded in younger respondents who are married. He attributes this fact to arising family conflicts that are associated with founding a family. Reference [10] found better WLB in older respondents. However, the results and conclusion of [28] are refuted by [7] who found that nurses having family did not report problems or imbalance compared to nurses living alone, suggesting that nurses living alone are exposed to more workload compared to nurses living with family. This result by [7] is supported by a more recent study by [10], which aimed to identify WLB among physicians. In this study, more frequent conflict was observed among single women. Only a negligible effect of marital status on WLB was found in our respondents (*ρ* = −0.021). Given the higher work–family conflict in younger and less experienced nurses, targeted support programs such as stress management training and mentorship by experienced colleagues are recommended to better equip this group to manage WLB challenges.

Surprisingly, this study did not find a statistically significant association between the number of children and WLB, although we expected one when selecting this variable. This finding contrasts with [23], who reported more frequent work-family conflicts among younger registered nurses due to childcare responsibilities. At the same time, studies [10, 29] agree that women report higher conflict compared to men. However, our ability to analyse gender differences was very limited due to the small number of male respondents in the sample (*n* = 5), which limits the reliability of these comparisons. Although we found a slightly positive relationship (0.1) for gender, suggesting that women may be slightly more affected by this factor than men, this finding should be interpreted with caution due to the unbalanced sample. Reference [29] suggests that this conflict could be due to the fact that women are both involved in the financial responsibilities of the family and provide an important role within family commitments. A possible explanation for the lack of association between the number of children and WLB in our study may be the influence of unmeasured mediating factors. For example, some respondents with children might have substantial support from their partners or access to external childcare services, which could mitigate the expected burden on WLB. In addition, individual differences in coping strategies may play an important role, as some individuals are better equipped to deal with the demands of both work and family life. These potential mediating variables should be included in future research to further understand the mechanisms influencing WLB in this context. To help mitigate the burden on nurses balancing family and work roles, enhancing family support systems and providing access to external childcare resources could be beneficial.

In addition, Ingstad and Haugan [30] found among nursing home employees that shift work is better for their work and personal lives than standard working hours. Even though shift work also means working on weekends and holidays, respondents reported better integration with their personal lives because of time off between shifts and the strict division of the 12-hour shift between work and family time, as well as less time associated with commuting. Better well-being was demonstrated than traditional working hours due to time flexibility. In contrast, night shift work was statistically significant in the study by [26] and negatively affected WLB. In this study, we found only a negligible relationship between shift work and WLB. Considering the Czech system of 12 h shifts combined with irregular schedules and overtime, setting up long-term shift planning and ensuring sufficient rest between shifts, including involving nurses in shift planning, could reduce negative impacts on WLB.

## 6. Conclusions

This study confirmed that the WLB among GNs is influenced by several individual and occupational factors, with particular attention to workplace type, education and professional experience. Although some expected predictors, such as number of children or shift operation, showed weaker associations, the findings underline that the burden of professional responsibilities often outweighs the demands of personal life.

Beyond statistical findings, this study points to clear practical implications for nursing management. Ensuring adequate staffing, offering flexibility in scheduling and supporting continuing education are essential strategies to mitigate WLB conflicts. Managers should particularly focus on nurses in demanding units, such as intensive care, who are at heightened risk of professional strain.

These results are also relevant in the broader European context, where similar challenges in healthcare staffing and retention are common. Strengthening WLB support systems could not only enhance job satisfaction but also contribute to stabilising the nursing workforce across countries facing similar demographic and workforce pressures.

Future research should explore tailored interventions that reflect the specific needs of healthcare systems in different cultural and organisational contexts, ensuring that strategies to improve WLB are both evidence-based and practically feasible.

## 7. Limitations of the Study

This study has several limitations that should be acknowledged. In the first place, it is a cross-sectional study; therefore, we cannot evaluate change over time. To gain a better understanding of the dynamics of WLB and to assess causality, **longitudinal research** is recommended. In addition, **intervention studies** are needed to identify effective strategies for supporting WLB among healthcare professionals.

The research focussed exclusively on GNs, with no input from practical nurses or other nonmedical staff. In addition, recruitment did not differentiate between nurses employed in public and private healthcare facilities, which may have influenced their perception of working conditions.

The analysis of gender differences is significantly limited due to the very small number of male respondents. This prevents reliable statistical comparisons between genders and limits the generalisability of the findings to the entire population of healthcare professionals.

Another limitation of this study is the exclusive use of online data collection, which may have introduced selection bias. Although the target group consisted of GNs who routinely use digital systems in healthcare, some subgroups—such as older nurses with lower digital literacy—may have been underrepresented. Additionally, nurses without internet access were unable to participate in the study. To increase the generalisability of the results, it is recommended to expand the sample of respondents and include practical nurses in the study. This would help to confirm the general validity of the findings for a wider group of nonmedical health professionals.

This study also did not include potential mediating variables (e.g., organisational culture, managerial support and individual coping strategies) that could help explain the mechanisms influencing WLB. Incorporating such factors in future research could provide a more comprehensive understanding of the underlying processes.

Furthermore, the lack of association between number of children or shift work and WLB was not further analysed to explore potential underlying reasons. Future research should address these issues in more detail, including possible mediating factors that might influence these relationships.

Finally, the study did not address cultural factors or assess the international generalisability of the findings. Given that WLB is shaped by cultural norms, societal expectations and healthcare system structures, future research should consider cross-cultural comparisons to better understand how these factors influence nurses' experiences globally.

## Figures and Tables

**Table 1:** Descriptive statistics of respondents (*N* = 200).

Variable	Mean	Standard deviation (SD)	Minimum	Median	Maximum
Gender	1.025	0.16	1	1	2
Age	45.1	10.6	21	47	65
Marital status	1.715	0.99	0	2	4
Number of children	1.29	0.95	0	1	4
Education	1.09	1.15	0	1	3
Additional education	0.3	0.76	0	0	5
Workload	1.24	0.64	1	1	4
Shift work	1.46	0.5	1	1	2
Length of experience	3.28	1.65	0	4	5
Workplace	2.38	1.04	1	2	4
WLB total	2.3	1	1	2	4

*Note:* Key: Gender: Factorised variable (1: females, 2: males). Age: Mean age is 45.1 years, SD 0.16. Marital status: Factorised variable (0—single, 1—living with partner, 2—married, 3—divorced, 4—widowed). Education: Factorised variable (0—secondary, 1—post-secondary vocational, 2—university bachelor's degree, 3—university master's degree). Additional education: Factorised variable (0—no, 1—university bachelor's degree, 2—university master's degree, 3—Ph.D., 4—post-secondary vocational, 5—PhDr.). Workload: Factorised variable (1—1.0, 2—0.5, 3—0.8, 4—0.3). Shift work: Factorised variable (1—yes, 2—no). Length of experience: Factorised variable (0—1–5 years, 1—6–10 years, 2—11–15 years, 3—16–20 years, 4—21–30 years, 5—31 and more years). Workplace: Factorised variable (1—standard, 2—intensive, 3—outpatient, 4—leading).

**Table 2:** Two-dimensional relationships between selected demographical and professional factors and work–life balance indicators (*N* = 200).

	I came home from work so tired that I was not able to do the things at home that were necessary.	Because of the amount of time I spent at work, it was difficult for me to fulfil family commitments.	Owing to work in the household I had to do, I arrived at work so tired that I was not able to work well.	I have found out that because of family commitments it is difficult for me to focus on work.
Mean total score (SD; median)	3 (0.78; 3)	2.8 (0.8; 3)	1.74 (0.82; 2)	1.76 (0.74; 2)

*Number of children mean score (SD, median)*				
None	3 (0.73, 3)	2.75 (0.87, 3)	1.94 (0.83, 2)	1.81 (0.83, 2)
One	3.04 (0.79, 3)	2.80 (0.69, 3)	1.59 (0.77, 1)	1.82 (0.71, 2)
Two	3.06 (0.89, 3)	2.78 (0.81, 3)	1.72 (0.8, 2)	1.73 (0.66, 2)
Three and more	2.5 (0.73, 2.5)	2.64 (0.72, 3)	1.64 (0.89, 1)	1.5 (0.82, 1)
Total *p* value	0.4777	0.8689	0.4549	0.4121

*Workplace mean score (SD, median)*				
Standard	3.02 (0.82, 3)	2.86 (0.67, 3)	1.76 (0.87, 2)	1.9 (0.91, 2)
Intensive	2.89 (0.81, 3)	2.66 (0.95, 3)	1.87 (0.79, 2)	1.61 (0.61, 2)
Outpatient	3.08 (0.75, 3)	2.75 (0.7, 3)	1.60 (0.74, 1)	1.85 (0.66, 2)
Leading position	3.03 (0.73, 3)	2.83 (0.76, 3)	1.75 (0.89, 1.5)	1.72 (0.69, 2)
Total *p* value	0.6031	0.0943^†^	0.5958	0.0886^†^

*Education mean score (SD, median)*				
Secondary	3.01 (0.81, 3)	2.72 (0.86, 3)	1.59 (0.81, 1)	1.7 (0.77, 2)
Post-secondary vocational	3 (0.88, 3)	2.85 (0.82, 3)	1.73 (0.81, 2)	1.88 (0.89, 2)
Bachelor's degree	3 (0.79, 3)	2.86 (0.77, 3)	2.04 (0.84, 2)	1.78 (0.67, 2)
Master's degree	2.97 (0.55, 3)	2.7 (0.53, 3)	1.7 (0.69, 2)	1.8 (0.54, 2)
Total *p* value	**0.027** ^∗^	0.3454	0.051^†^	0.1444

*Length of experience mean score (SD, median)*				
1–10 years	3.03 (0.75, 3)	2.78 (0.84, 3)	1.95 (0.84, 2)	2.05 (0.93, 2)
11–20 years	2.93 (0.78, 3)	2.75 (0.71, 3)	1.82 (0.83, 2)	1.70 (0.69, 2)
21–30 years	3.02 (0.81, 3)	2.79 (0.79, 3)	1.76 (0.84, 2)	1.74 (0.70, 2)
31 and more years	3.02 (0.76, 3)	2.75 (0.82, 3)	1.51 (0.72, 1)	1.62 (0.59, 2)
Total *p* value	0.9936	0.9741	0.5284	0.1700

Abbreviation: SD, standard deviation.

^†^> 0.05 *p*< 0.10 (marginal significance).

^∗^ *p* < 0.05.

**Table 3:** Level of conflict between selected demographical and professional factors (*N* = 200).

	High level of conflict	Medium level of conflict	Low level of conflict
Mean total score (SD; median)	14.11 (1.2, 14)	10.1 (1.03, 10)	7.01 (1.08, 7)

*Number of children mean score (SD; median)*			
None	14.17 (1.34; 13.5)	10.26 (1.07, 10)	7.1 (1.04, 7.5)
One	14.7 (1.25; 15)	10.17 (1.05, 10)	7 (1.03, 7)
Two	13.63 (0.7, 13.5)	9.96 (0.98, 10)	6.93 (1.09; 7)
Three and more	16 (0; 16)	10 (0.82, 10)	7 (1.18, 7)

*Workplace mean score (SD, median)*			
Standard	14.8 (1.47; 16)	10.33 (1.07; 10)	7.15 (0.96; 7.5)
Intensive	14 (1.15; 13.5)	9.95 (0.1; 10)	7.17 (1.01; 10)
Outpatient	13.3 (0.47; 13)	10.08 (1.16; 10)	6.72 (1.25; 7)
Leading position	14 (0.82; 14)	10.39 (1.11; 10)	7.27 (0.93; 8)

*Education mean score (SD; median)*			
Secondary	14 (1.25; 13)	10.1 (1.39; 10)	7.02 (1.1; 7)
Post-secondary vocational	14.5 (1.5; 14.5)	10.13 (1.02; 10)	7.22 (1.22; 8)
Bachelor's degree	14.17 (1.07; 14)	10 (1.61; 10)	6.93 (1.16; 7)
Master's degree	0	10.15 (0.85; 10)	7.2 (0.7; 7)

*Length of experience mean score (SD; median)*			
1–10 years	14.17 (1.36; 13.5)	10.22 (1.13; 10)	7.23 (0.9; 8)
11–20 years	14.33 (1.7; 14)	9.92 (1; 10)	6.93 (1.12; 7)
21–30 years	14.17 (1.21; 14)	10.44 (1.09; 10)	6.96 (1.09; 7)
31 and more years	13.67 (0.47; 14)	9.79 (0.76; 10)	7 (1.07; 7)

Abbreviation: SD, standard deviation.

**Table 4:** Statistically and marginally significant differences in work–life balance (*N* = 200).

Variable	Item (WLB)	*p*
*Workplace*		
Standard	Item 4	0.0229^∗^
Intensive	Item 2	0.0051^∗∗^

*Number of children*		
Three and more	Item 1	0.0934^†^

*Education*		
Secondary	Item 1	0.0072^∗∗^
Secondary	Item 3	0.0141^∗^
Master's degree	Item 1	0.0556^†^
Master's degree	Item 2	0.0571^†^

*Length of experience*		
1–10 years	Item 4	0.01^∗^
31 and more years	Item 3	0.0939^†^

^†^⟶0.05 < *p* < 0.10 (marginal significance).

^∗^ *p* < 0.05.

^∗∗^ *p* < 0.01.

**Table 5:** Linear regression model for predictors of work–life balance score (*N* = 200).

Predictors	*B*	SE	95% Cl	*p*
Age	−0.007	0.0144	(−0.036; 0.022)	0.624
Children	0.128	0.2552	(−0.386; 0.641)	0.619
Education	−0.031	0.2573	(−0.549; 0.486)	0.903
Marital status	−0.023	0.2414	(−0.509; 0.463)	0.924
Length of experience	0.094	0.2895	(−0.488; 0.676)	0.747
Shift operation	0.019	0.2310	(−0.446; 0.483)	0.936
Workplace	0.345	0.2537	(−0.165; 0.856)	0.180

*Note: B*, regression coefficient; *p*, statistical significance.

Abbreviations: Cl, confidence interval; SE, standard error.

**Table 6:** Correlation between work–life balance in the comparison of all variables (*N* = 200).

Characteristics (Spearman's correlation coefficients)	I came home from work so tired that I was not able to do the things at home that were necessary.	Because of the amount of time I spent at work, it was difficult for me to fulfil family commitments.	Owing to work in the household I had to do, I arrived at work so tired that I was not able to work well.	I have found out that because of family commitments it is difficult for me to focus on work.
Age	0.016	−0.022	−0.178	−0.101
Gender	0.036	−0.002	0.1	0.064
Marital status	−0.034	−0.021	−0.024	0.058
Number of children	−0.047	−0.016	−0.106	−0.081
Education	−0.015	0.023	0.184	0.1
Additional education	−0.013	0.04	0.087	0.075
Workload	−0.003	−0.055	0.08	−0.033
Shift operation	0.037	−0.125	−0.118	−0.025
Length of experience	0.012	−0.007	−0.192	−0.128
Workplace	0.012	−0.007	−0.192	0.354

## Data Availability

The data that support the findings of this study are available from the corresponding author upon reasonable request.

## Supporting Information

The following supporting information was submitted with the manuscript.

*STROBE checklist*—A checklist according to the STROBE (Strengthening the Reporting of Observational Studies in Epidemiology) guidelines that confirms that the manuscript meets the requirements for structure and transparency of observational study reports.
